# Suitability of radiochromic films for dosimetry of low energy X‐rays

**DOI:** 10.1120/jacmp.v10i4.2957

**Published:** 2009-09-02

**Authors:** Martin A Ebert, Ali H Asad, Salim A Siddiqui

**Affiliations:** ^1^ Department of Radiation Oncology Sir Charles Gairdner Hospital Nedlands Western Australia 6009; ^2^ School of Physics University of Western Australia Crawley Western Australia 6009; ^3^ Department of Imaging and Applied Physics Curtin University of Technology Bentley Western Australia 6102

**Keywords:** radiochromic flm, intraoperative radiotherapy, kV dosimetry

## Abstract

This study examined the response characteristics of three commercially available radiochromic films when exposed to low energy (50 kVp) X‐rays. The aim was to evaluate the films for potential use in 2D dosimetry for a low‐kV intraoperative radiotherapy (IORT) device known as the ‘Intrabeam’. Dose‐response relationships were obtained for Gafchromic EBT, XR‐RV2, and XR‐QA film in water at several distances from the Intrabeam device. It was found that the dose rates from the source were excessive for use of the XR‐QA film, and that all three film types showed significant energy dependence within the limits of measurement uncertainty. Basic modeling of primary X‐ray spectra indicated large changes in the lower energy components with distance from the source, and it is hypothesized that the changes in film response are a result of changes in film energy response. This is in contrast to previous studies indicating less or negligible energy response. All films showed non‐linearity in response over the ranges examined. These results imply significant limitations for the use of these films for low‐kV dosimetry.

PACS number: 87.53.Bn

## I. INTRODUCTION

Radiochromic film has been investigated extensively for dosimetry in radiation medicine.^(^ ^1^ ^,^ ^2^ ^)^ The energy response of multiple film types has been studied by several authors.^(^ ^3^ ^–^ ^9^ ^)^ These investigations reveal sensitivity changes with beam quality, with recently‐developed films (such as the ‘EBT’ and ‘XR’ series) displaying variations of several percent over energy ranges expected in a single measurement, including measurements in the superficial X‐ray energy range.^(^ ^4^ ^,^ ^5^ ^)^ Rampado et al.^(10)^ found much larger variations in response with energy for XR‐QA film, particularly in the low‐to‐medium X‐ray energy range (28–140 kVp).

Radiochromic film has multiple advantages over alternative dosimeters for dosimetry of low‐kV X‐rays. Its high spatial resolution is far superior to that of ionization chambers and thermoluminescent dosimeters (TLDs).^(7)^ Compared to radiographic film, radiochromic film offers greater X‐ray sensitivity, reduced processing and handling demands, and an ability to be used in water without waterproof encapsulation.

In this work, an intraoperative radiotherapy (IORT) device was used as a radiation source. This device produces 50 kVp X‐rays from an effective point‐source which can be positioned *in situ* during therapy. The combination of rapid X‐ray divergence, attenuation and spectral hardening in tissue/water makes dosimetry a challenge. Initial measurements of 2D distributions in water which we undertook with EBT film (International Specialty Products, Wayne NJ) were found to differ significantly from measurements made with an ionisation chamber and with theoretical distributions.^(11)^ As a result, the investigation presented here was commenced with the aim of investigating alternative film types to determine whether the response of the films changes with distance from the source, and whether changes in response could be attributed to changes in X‐ray spectra.

## II. MATERIALS AND METHODS

### A. X‐ray source

The IORT ‘Intrabeam’ device [the ‘Photon Radiotherapy System’ (PRS) developed by Photoelectron Corporation (Lexington, MA), currently manufactured by Carl Zeiss AG (Germany)] was used in this study as the X‐ray source. Detailed descriptions of this device have been provided elsewhere.^(^ ^12^ ^–^ ^14^ ^)^ Use of this device at the Sir Charles Gairdner Hospital is with the 50 kVp beam only and, as such, all measurements reported here are for the 50 kVp beam.

### B. Output linearity measurements

The Intrabeam was used in a mode specifically designed for beam dosimetry, whereby total output is controlled by a timer. Linearity of output with timer setting was investigated using a PTW type M23343 soft X‐ray parallel plate chamber (PTW‐Freiburg), connected to a NE 2670 Farmer dosimeter (Thermo Scientific, Waltham MA). The chamber was placed in a Perspex holder with its front window against the Intrabeam to which was attached a 30 mm diameter applicator.

### C. Distance‐dose data and uncertainty

Distance‐dose data (dose rate measured with distance from the Intrabeam source) was measured using the thin‐window chamber discussed above, with corrections for spectral variations made according to the protocol of the IPEM.^(^ ^15^ ^,^ ^16^ ^)^ Note that distances mentioned in this paper are from the outside tip of the probe, not from the effective source position which is approximately at the centre of curvature of the probe tip (with 1.6 mm radius).^(13)^ Uncertainties in doses in this report have been evaluated from measured data according to an uncertainty in dosimeter position of ±1mm, which translates to dose uncertainties of up to 45% at 5 mm from the X‐ray source, and 13% at 30 mm from the source. This is combined in quadrature with contributions due to timer error.^(12)^

### D. Radiochromic film

Three types of radiochromic film, manufactured by ISP (International Specialty Products, Wayne NJ) were investigated. Their reported characteristics are shown in Table 1. The EBT type was specifically designed for use in external beam radiotherapy applications and for analysis using light transmission measurements. The XR‐QA film uses light refection for analysis. It has the same chemical composition of EBT film, but with high atomic number halides added to the sensitive layer to increase photoelectron interaction. The intention is to make the film suitably responsive to lower energy (<200kVp) X‐rays^(17)^ for radiology quality assurance. The XR‐RV2 film, specifically designed for tracking spatial exposure patterns during fluoroscopy procedures, also produces an image that must be read out with light refection.

**Table 1:** Film types examined with reported characteristics.

*Film Type*	*Recommended energy range* ^a^	*Recommended dose range* ^a^	*Chemical composition* ^a^	*Structural layers* ^a^
EBT	kV–MV	0.01–8 Gy	C 42.3% H 39.7% O 16.2% N 1.1% Li 0.3% Cl 0.3% $(Z_{\text{eff}}\;6.98\%$)	Clear polyester (97 μm) Active layer (17 μm) Surface layer (6 μm) Active Layer (17 μm) Clear polyester (97 μm)
XR‐RV2	30 kV–30 MV^a^	0.01–10 Gy	Not available	Clear yellow‐dyed polyester (97 μm) Adhesive Surface layer (3 μm) Active layer (17 μm) Opaque white polyester (97 μm)
XR‐QA	20 kVp–200 kVp	0.001–0.2 Gy^a^	As per EBT with high Z halides added to sensitive layer^(17)^	Clear polyester (97 μm) Active layer (25 μm) Surface layer (5 μm) Surface layer (5 μm) Active layer (25 μm) Opaque white polyester (97 μm)

^a^ Information from ISP website, http://www.ispcorp.com/

### E. Film handling & optical density reading

Film pieces were attached to a backing material (transparency film for EBT and plain white paper for XR‐RV2 and XR‐QA) and scanned on a Microtek Scanmaker 9800XL scanner (Microtek, Cerritos CA). For transmission measurements, a backlight attached to the scanner was used. Scanned images were saved in 24‐bit TIFF format. Two separate observers (MAE and AA) utilized ImageJ (http://rsb.info.nih.gov/ij/) to evaluate the red colour channel^(6)^ of each image.

Recommendations for the handling and scanning of radiochromic films presented in the literature were followed.^(^ ^1^ ^,^ ^10^ ^,^ ^18^ ^–^ ^22^ ^,^ ^23^ ^)^ Specifically, the following procedures were adhered to:

- Films were stored dry, in an air‐conditioned environment and in lightproof envelopes.
- The time between irradiation and scanning was at least 24 hours.
- A consistent orientation of the original film was maintained for all scans. It has previously been shown^(18)^ that scanning orientation can influence measured optical density due to alignment of particles in a film's active layer, influencing light scattering during scanning.
- An attempt to account for variations in scanner readings with spatial position^(^ ^20^ ^,^ ^21^ ^,^ ^22^ ^,^ ^24^ ^)^ was made by distributing repeat films for measurements randomly across the central 50% width of the scanner surface. Repeat scans of films in different regions of the scan area showed variations in pixel intensity of less than 1%. Sample density films were always included in scans to reference intensity levels.
- The scanner was warmed up and prescanned.
- Reference exposed and unexposed film pieces were scanned at the same time as measurement films, to normalize pixel values between scans^(22)^ and to establish base‐plus‐fog levels.

The dose response of the films was quantified using a measure of optical density (OD),^(^ ^5^ ^,^ ^20^ ^,^ ^25^ ^,^ ^26^ ^–^ ^29^ ^)^ relating a reference light intensity, $I_{0}$, to that transmitted through (for EBT film) or reflected from (for XR‐QA and XR‐RV2 films) the film, *I*. OD is defined by:

(1) $$OD={\text{log}}_{10}\left(\frac{I_{0}}{I}\right)$$

The Net Optical Density (NOD) for transmission through/reflection from any piece of exposed film is given by subtraction of the optical density for an unexposed film piece:

(2) $$\text{NOD}={\text{log}}_{10}\left(\frac{I_{0}}{I_{e}}\right)-{\text{log}}_{10}\left(\frac{I_{0}}{I_{u}}\right)={\text{log}}_{10}\left(\frac{I_{u}}{I_{e}}\right)$$

where $I_{o}$ is the intensity of the incident light beam, $I_{e}$ is the intensity of the light transmitted through/reflected from the exposed film piece, and $I_{u}$ is the intensity transmitted through/reflected from an unexposed film piece (where ‘intensity’ is determined according to pixel value in the scanned image). This process inherently convolves the relationship between light reflection/transmission and pixel value into the film dose response relationship.

The following potential sources of variations in response measurements were also examined for the three film types:

- The time the film was in water – As this study involved using film submerged in water for times varying from approximately one minute to one hour, the influence of submersion on exposed and unexposed films was investigated.
- Dose rate – Pieces of each film type were exposed, in air, to 50 kVp X‐rays from the Intrabeam device at three distances from the X‐ray source, with distances selected to achieve a variation in dose rate of up to two orders of magnitude. The M23343 ionisation chamber was used to determine the time required at each distance to achieve the same dose (selected to achieve a NOD of approximately 0.5).

### F. Film exposures – dose response

A sheet of each of the three films was cut into multiple squares 20mm×20mm. The pieces were marked for orientation, mixed to reduce the influence of spatial non‐uniformities, and numbered. Individual pieces were taped to a block of solid water (GAMMEX RMI, Middleton WI), and placed in a water tank at fixed distances of either 5 mm, 15 mm or 30 mm from the tip of the Intrabeam probe (without an applicator attached), with the centre of the film aligned with the axis of the Intrabeam probe. At each timer (dose) setting, three films from each sheet were exposed.

Dose rates for exposures were 14.3 Gy/min at 5 mm, 0.92 Gy/min at 15 mm, and 0.11 Gy/min at 30 mm distance in water from the outside tip of the Intrabeam probe. Exposure times were selected based on the reported dose range for each film type. For the EBT and XR‐RV2 films, exposures of between 0.05 and 1 minutes were required at 5 mm distance, and between 5 and 50 minutes at 30 mm. For the XR‐QA film, exposures ranged from between 0.01 and 0.1 minutes at 5 mm, to between 0.5 and 5 minutes at 30 mm. The timer setting resolution is 0.01 minutes.

Due to the near point‐source nature of the Intrabeam emission, the pixel variations on each film piece followed a concave pattern. Minimum pixel values were obtained by examining histograms of the central regions of each film piece following smoothing by 3×3 pixel averaging.

### G. Spectrum modeling

As the energy response of the film could contribute to changes in film response with distance from the source, we examined the X‐ray spectrum at each measurement depth. The X‐ray spectrum for the 50 kVp beam from the Intrabeam was measured using a cadmium zinc telluride detector (CdZTe‐type XR‐100T‐CZT, Amptek Inc, Bedford MA) connected to a MCA8000A multichannel analyzer (Ampek Inc, Bedford MA). A 1 mm diameter pinhole in a 2 mm thick lead sheet was used to reduce beam intensity.^(11)^ Changes in the primary X‐ray spectra with distance in water (at 5 mm, 15 mm and 30 mm) were calculated using exponential and inverse‐square attenuation, with linear attenuation coefficients obtained using data from ICRU Report 46^(30)^ and the program XCOM^(31)^ (available online at http://physics.nist.gov/PhysRefData/Xcom/Text/XCOM.html).

This very simple method of calculation for primary spectrum variations has previously been shown to be effective for the calculation of dose distributions from the Intrabeam source.^(11)^

## III. RESULTS

### A. Output linearity

Dose output was found to be proportional to timer setting. A regression line through the data has $r^{2}=1.000$ with less then 1.5% discrepancy from linearity across all measurements.

### B. Factors influencing film response

- The time the film was in water – The time the film was submerged in water was found to have no significant relationship to measured optical density over the range 0.05 to 50 minutes.
- Dose rate – Although a small variation in response with dose rate has previously been reported for EBT film,^(32)^ a significant dose rate effect was not observed within the error of our measurements over the range of dose rates utilized in response measurements.

### C. Dose response

**Figure 1:**
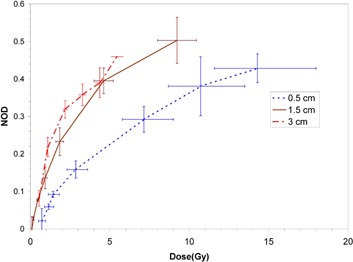
Measured dose response curves for EBT film at the three measurement source‐to‐film distances in water. Vertical error bars are based on the standard deviation of three separate measurements, and horizontal error bars on an assumed 1.0 mm uncertainty in establishment of the source‐to‐detector distance and 0.01 minute timer error.

**Figure 2:**
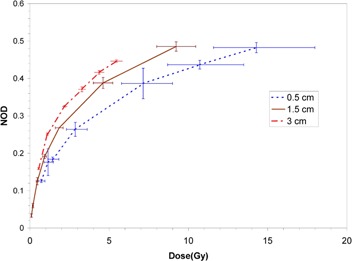
Measured dose response curves for XR‐RV2 film at the three measurement source‐to‐film distances in water. Vertical error bars are based on the standard deviation of three separate measurements, and horizontal error bars on an assumed 1.0 mm uncertainty in establishment of the source‐to‐detector distance and 0.01 minute timer error.

**Figure 3:**
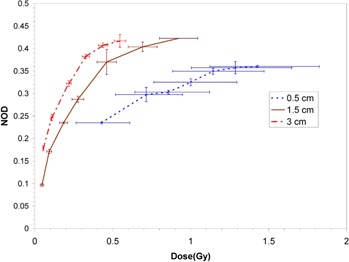
Measured dose response curves for XR‐QA film at the three measurement source‐to‐film distances in water. Vertical error bars are based on the standard deviation of three separate measurements, and horizontal error bars on an assumed 1.0 mm uncertainty in establishment of the source‐to‐detector distance and 0.01 minute timer error.

Dose response curves at the three measurement source‐to‐film distances for the EBT, XR‐RV2 and XR‐QA films are shown in Fig. 1, Fig. 2, and Fig. 3, respectively.

### D. X‐ray spectra

**Figure 4:**
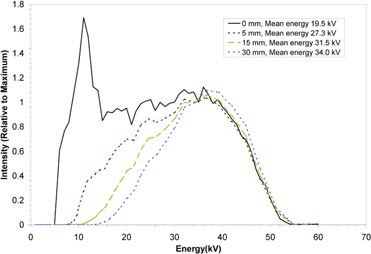
Primary beam X‐ray spectra with distance through water from the Intrabeam source. All spectra have been normalized to the intensity at 35 kV. The mean X‐ray energy at each distance is indicated in the legend.

Figure 4 shows the primary X‐ray spectra at relevant distances in water from the Intrabeam source, together with the mean energy at each distance. For comparison, the in air measured spectrum (‘0 mm’) is also shown.

## IV. DISCUSSION

The size of horizontal error bars in Figs. 1, 2 and 3 reflect the steepness of the dose gradient with distance from the Intrabeam source. This gradient makes precise dosimetry a challenge. An uncertainty in film positioning of ±1mm is certainly generous, as the film could be positioned quite precisely and reproducibly relative to the Intrabeam effective source position. For the XR‐QA film at 5 mm distance, the error due to distance uncertainty is of the order of that due to timer uncertainty.^(12)^ Dose rates in the water could not be reduced experimentally without perturbing the beam spectrum (achievable variations in device current would be insufficient to allow large changes in dose rate). As a result, the XR‐QA film is difficult to use with this X‐ray source and would not be recommended for related dosimetric purposes.

All three film types demonstrated significant variation in response at the three distances considered, with a consistent increase in sensitivity (NOD at the same dose) with increasing distance from the X‐ray source (corresponding to increasing mean energy of the primary X‐rays). All film types display non‐linearity in response at all distances. For the EBT and XR‐RV2 films, saturation of optical density was not reached at the shorter distances since this would have required excessively impractical irradiation times.

Although energy dependence of film response has previously been demonstrated, the energy dependence of EBT film has previously been reported to be fairly minimal.^(^ ^5^ ^,^ ^8^ ^)^ In particular, Chiu‐Tsao et al.^(6)^ reported no variation in response for EBT (within measurement uncertainty) range over the energy range from P103d (21 keV) to a 6 MV accelerator beam. This included a comparison for response between P103d and I125 (28 keV) where a rapid modulation in response, as seen in the results presented here, could have been observed but was not. The changes in response corresponding to small changes in energy observed here are therefore confounding.

The sensitivity change of the XR‐QA film is also inconsistent with the report of Chiu‐Tsao et al.^(6)^ Although they report a significant change in response between kV and MV X‐rays, the small changes they observed in response between P103d and I125 are inconsistent with the results obtained here. Other previous reports have identified a significant energy response for XR‐type films,^(^ ^4^ ^,^ ^33^ ^)^ with response rising rapidly at low energies up to a maximum at a mean X‐ray energy of around 54 kV for XRCT. This is consistent with the results presented here. Comparison of percentage differences in response at any particular dose is not possible for the results presented here as there is no common dose value that covers all response curves over all film types.

A principal difference between the irradiation at each distance was the rate of dose delivery. A slight dose rate dependence has previously been reported for EBT film.^(32)^ Although we attempted to assess this by using films exposed to the Intrabeam source in air (thereby reducing the influence of energy changes), we did not identify any consistent variation in response with dose rate within the standard deviation of resulting optical densities. As such, it is unlikely that dose rate effects contribute to the substantial differences we observed in film response at different source‐to‐detector distances.

Radiochromic films would, for practical reasons, provide an excellent medium for rapidly generating two‐dimensional dosimetric data for the intraoperative source being investigated. However, the combination of rapid energy change with distance in water with the energy dependencies found here suggest a level of accuracy that is well surpassed by using a low‐energy ionization chamber. In contrast however, Armoogum and Watson^(34)^ investigated the Gafchromic XR Type‐R film with the same type of intraoperative source and found an energy response that was sufficiently small (less then 6% across the range 30 kVp to 50 kVp) to enable use of the film for dosimetric intercomparison between devices. They irradiated films at a fixed source‐to‐film distance in their experiments and varied energy by adjusting the kV of the source. This was not attempted in the study presented here due to a lack of dosimetric data for the device at voltages other then 50 kV.

## V. CONCLUSIONS

This study revealed significant changes in response of several radiochromic film types for small changes in X‐ray characteristics from a low energy source. This is in contrast to previous studies which have reported smaller or negligible energy dependence for similar film types. The use of radiochromic films for measuring spatial dose distributions at low X‐ray energies should be undertaken with caution. Although these films provide a convenient means to quickly measure large amounts of spatial dose information, the magnitudes of response variation identified for the film types investigated here suggests that they are not suitable for obtaining accurate quantitative information.

## ACKNOWLEDGEMENTS

Many thanks to Peter Lanzon and Setayesh Behin‐Ain for support with measurements. We are very grateful to CMS Alphatech for providing samples of radiochromic films. This work was supported in part by grant 393703 from the National Health and Medical Research Council of Australia.
